# Chitosan-Based Anti-Oxidation Delivery Nano-Platform: Applications in the Encapsulation of DHA-Enriched Fish Oil

**DOI:** 10.3390/md19080470

**Published:** 2021-08-22

**Authors:** Po-Kai Chang, Ming-Fong Tsai, Chun-Yung Huang, Chien-Liang Lee, Chitsan Lin, Chwen-Jen Shieh, Chia-Hung Kuo

**Affiliations:** 1 Department of Seafood Science, National Kaohsiung University of Science and Technology, Kaohsiung 811, Taiwan; encorek30@gmail.com (P.-K.C.); l38982079@gmail.com (M.-F.T.); cyhuang@nkust.edu.tw (C.-Y.H.); 2 Department of Chemical and Materials Engineering, National Kaohsiung University of Science and Technology, Kaohsiung 811, Taiwan; cl_lee@nkust.edu.tw; 3 Department of Marine Environmental Engineering, National Kaohsiung University of Science and Technology, Kaohsiung 811, Taiwan; ctlin@nkust.edu.tw; 4 Biotechnology Center, National Chung Hsing University, Taichung 402, Taiwan; cjshieh@nchu.edu.tw; 5 Center for Aquatic Products Inspection Service, National Kaohsiung University of Science and Technology, Kaohsiung 811, Taiwan

**Keywords:** powdered fish oil, docosahexaenoic acid, chitosan nanoparticles, encapsulation efficiency, loading capacity, TGA, FTIR, oxidative stability

## Abstract

Refined cobia liver oil is a nutritional supplement (CBLO) that is rich in polyunsaturated fatty acids (PUFAs), such as DHA and EPA; however, PUFAs are prone to oxidation. In this study, the fabrication of chitosan-TPP-encapsulated CBLO nanoparticles (CS@CBLO NPs) was achieved by a two-step method, including emulsification and the ionic gelation of chitosan with sodium tripolyphosphate (TPP). The obtained nanoparticles were inspected by dynamic light scattering (DLS) and showed a positively charged surface with a z-average diameter of between 174 and 456 nm. Thermogravimetric analysis (TGA) results showed the three-stage weight loss trends contributing to the water evaporation, chitosan decomposition, and CBLO decomposition. The loading capacity (LC) and encapsulation efficiency (EE) of the CBLO loading in CS@CBLO NPs were 17.77–33.43% and 25.93–50.27%, respectively. The successful encapsulation of CBLO in CS@CBLO NPs was also confirmed by the Fourier transform infrared (FTIR) spectroscopy and X-ray diffraction (XRD) techniques. The oxidative stability of CBLO and CS@CBLO NPs was monitored by FTIR. As compared to CBLO, CS@CBLO NPs showed less oxidation with a lower generation of hydroperoxides and secondary oxidation products after four weeks of storage. CS@CBLO NPs are composed of two ingredients that are beneficial for health, chitosan and fish oil in a nano powdered fish oil form, with an excellent oxidative stability that will enhance its usage in the functional food and pharmaceutical industries.

## 1. Introduction

Cobia (*Rachycentron canadum*) are a medium-sized migratory carnivorous fish that is widely distributed in tropical marine locations. Cobia has a high economic value and is a fish species that is popular for commercial aquaculture, with a global production of ~43,000 tons per year [1]. Cobia are mainly processed into fillets, with by-products of around 65% of the total weight generated [2,3]. Cobia liver is one of these waste products, accounting for 4% of the total weight and containing 46~48% fat; it is a potential raw material that could be used for producing fish oil. Cobia liver oil (CBLO) is rich in polyunsaturated fatty acids (PUFAs), such as DHA (Docosahexaenoicacid) and EPA (Eicosapentaenoic acid) [4]. DHA and EPA have been reported to possess anti-inflammatory and antidiabetic activities and can reduce the risk of cardiovascular disease, cancer, and Alzheimer’s disease [5,6,7]. However, DHA and EPA are unstable due to the risk of oxidation, as the rate of oxidation increases with the number of double bonds in a fatty acid [8,9]. Oxidation exerts negative effects on flavor and nutritional value, thereby limiting the application of PUFA-enriched fish oil in nutritional supplements.

Reducing exposure to oxygen is one way to avoid the oxidation of fish oils. The encapsulation of lipophilic compounds in biocompatible materials with a nano-technique is a feasible means for achieving this purpose [10,11]. As a result, the encapsulation of CBLO can improve its stability, helping to avoid irradiation, oxidation, and thermal degradation while reducing the fishy smell. Chitosan, a cationic polysaccharide with an outstanding biocompatibility and biodegradability, is widely applied in the field of biomedicine [12,13]. The structure of chitosan comprises β(1,4)-linked d-glucosamine and *N*-acetyl-d-glucosamine; the pKa value of the primary amine is around 6.5, depending on the degree of *N*-deacetylation [14]. Chitosan is pH-sensitive due to the amino groups of d-glucosamine possessing a positive charge at pHs below 6, which cause chitosan to become a water-soluble cationic biopolymer. However, chitosan is insoluble in physiological conditions at neutral pH. Such characteristics make chitosan suitable for the encapsulation and delivery of lipophilic compounds [15,16]. In particular, chitosan exhibits many health benefits, helping one to resist ulcers, lowering cholesterol, reducing blood lipids, and helping in the prevention of coeliac disease [17,18]; based on the above benefits, chitosan is a suitable wall material that can be used for the encapsulation of drugs [19] and can help to achieving the purpose of controlled delivery [20].

Recently, several nanoencapsulation processes utilizing chitosan have been developed, such as ionic gelation [21], electrospinning [22], emulsion-homogenization [23], self-assembly [24], and antisolvent precipitation [25]. The ionic gelation method is the favorite among these, as it is non-toxic, non-solvent, and easily controllable. The ionic gelation technique is based on the electrostatic interaction between the positively charged amino groups of chitosan and the negatively charged groups of anions (such as sodium tripolyphosphate, TPP) to form a safety component, CS-TPP nanoparticles (CS NPs) [26,27]. CS NPs have been used for loading insulin and also applied in diabetes therapy [28]. Moreover, several food bioactive ingredients have been encapsulated in CS NPs, including curcumin [29], flavonoids [30], lutein [31], polyphenols [32], resveratrol [33], and vitamins (B9, B12, and C) [34]. Fish oils are rich in ω-3 PUFAs, which are highly prone to oxidation due to their higher content of unsaturated fatty acid undergoing lipid oxidation. The encapsulation of fish oil can protect unsaturated fatty acids against oxidation and help to avoid unwanted reactions [35]. However, as there is little literature available regarding CS NPs encapsulated fish oil, the effect of encapsulation on preventing the oxidation of ω-3 PUFAs is worthy of study.

In this study, the encapsulation of CBLO containing ω-3 PUFAs by chitosan at the nano-scale was explored. A two-step procedure (emulsification and ionic gelation) was performed to fabricate CS-TPP-encapsulated CBLO nanoparticles (CS@CBLO NPs). The characterizations of CS@CBLO NPs were investigated by scanning electron microscopy (SEM), dynamic light scattering (DLS), thermogravimetric analysis (TGA), X-ray diffraction (XRD), and Fourier transform infrared spectroscopy (FTIR). The effects of the initial CBLO content on the encapsulation efficiency (EE) and loading capacity (LC) were also investigated. Finally, the effect of CS@CBLO NPs on the oxidative stability of CBLO was evaluated.

## 2. Results and Discussion

### 2.1. Shape and Size of CS-TPP Encapsulated CBLO Nanoparticles

**Figure 1:**
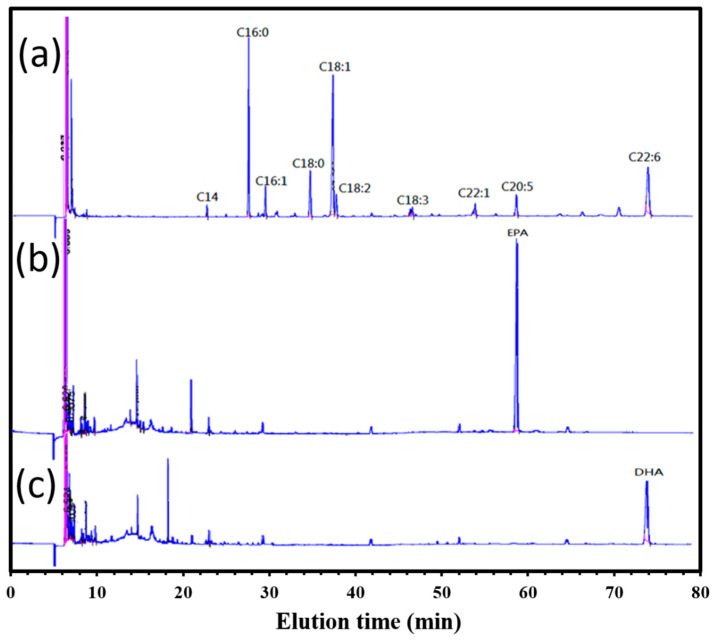
GC analysis of (**a**) the fatty acid profile of CBLO, (**b**) EPA standard, and (**c**) DHA standard.

Refined cobia liver oil (CBLO) with an acid value of 0.15 mg KOH g^−1^ was used as the core material for encapsulation. The fatty acid profile of the CBLO is presented in Figure 1. The CBLO contained 24.52% total ω-3 PUFAs (18.85% DHA, 4.25% EPA, and 1.42% α-linolenic acid).

**Scheme 1:**
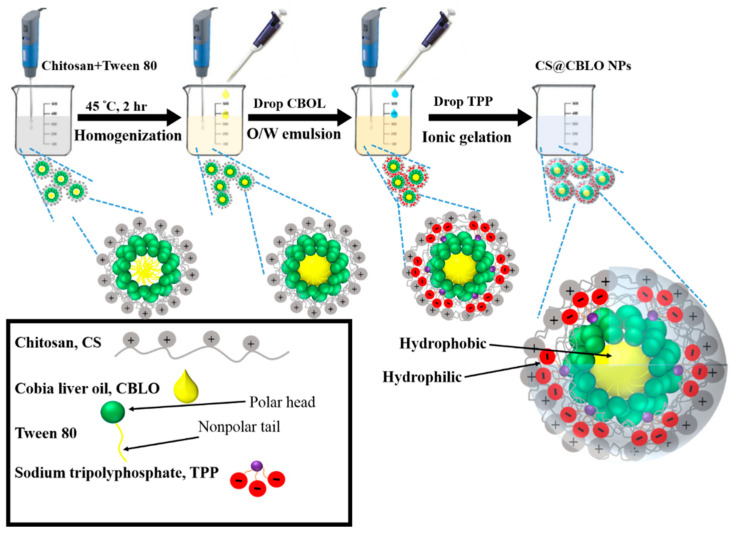
The two-step method used for fabrication of CS@CBLO NPs via emulsification and ionic gelation.

**Figure 2:**
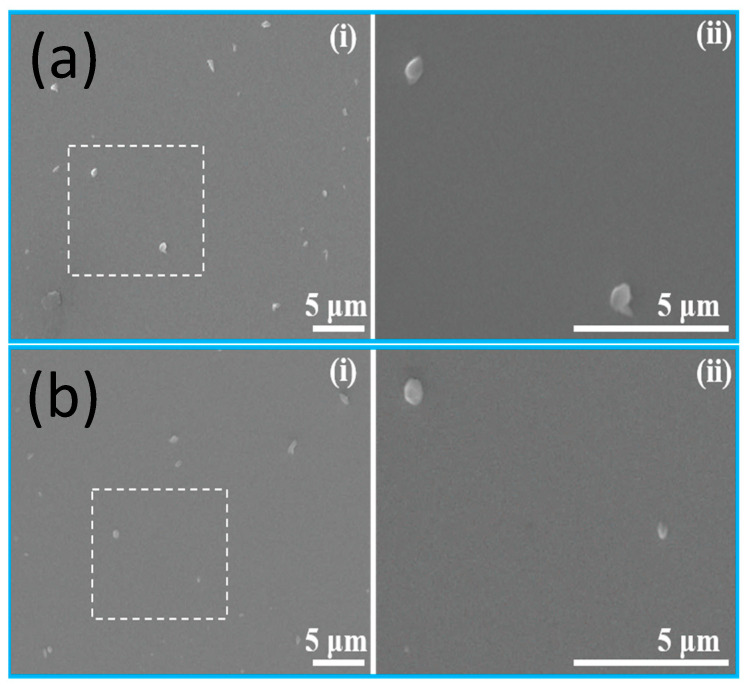
SEM images of (**a**) CS NPs and (**b**) CS@CBLO NPs (weight ratio of CBLO:chitosan at 1:1) at 2 kV. (**i**) and (**ii**) are the measured magnification at 5000× and 15,000×, respectively.

The two-step method used for the fabrication of CS@CBLO NPs is illustrated in Scheme 1. The first step was emulsification; the chitosan was treated with the surfactant reagent Tween 80 for entrapment. CBLO gained an oil-in-water micelle structure. The second step was the solidification of the micelles by the ionic gelation of chitosan with TPP to form CS@CBLO NPs. The morphology of the obtained particles was observed by SEM. As shown in Figure 2a,b, both CS NPs and CS@CBLO NPs exhibit a spherical shape and nanosized structure. The particle sizes of CS NPs and CS@CBLO NPs measured by SEM were 726 ± 136 nm and 347 ± 118 nm, respectively. The smaller particle size of CS@CBLO NPs was due to the homogenization dispersing the hydrophobic CBLO in the solution and forming micelles with the surfactant. While the CS NPs were formed by the electrostatic interaction of chitosan and TPP, the CS@CBLO NPs were formed by the ionic gelation of chitosan absorbed on the micelle. The hydrophobic CBLO in the micelle reduced the aggregation of chitosan on the micelle surface and thus produced smaller particles.

**Figure 3:**
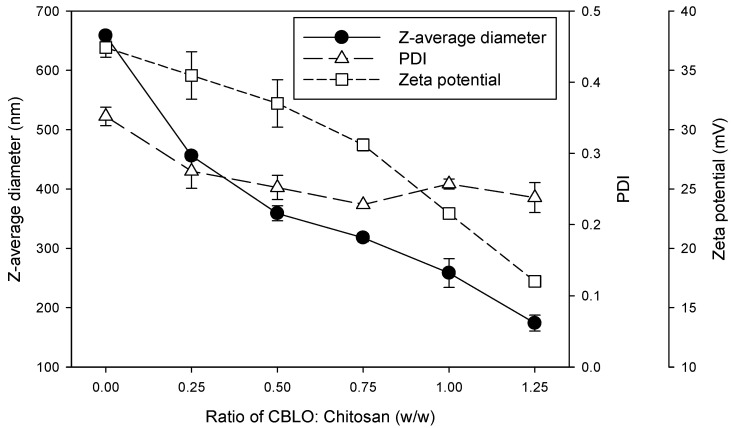
Z-average diameter, PDI and zeta potential of CS NPs and CS@CBLO NPs with different weight ratios of CBLO to chitosan. Data are the mean + standard deviation (*n* = 3).

The z-average diameter, PDI, and Zeta potential of CS NPs and CS@CBLO NPs were examined by DLS. Figure 3 shows that the z-average diameter of CS NPs is ~658 nm, while the z-average diameter of CS@CBLO NPs is between 174 and 456 nm. The DLS analysis was carried out in the aqueous environment; thus, the particle size would depend on the extent of the aggregation or swelling of the chitosan. Since the CBLO was entrapped inside the CS@CBLO NPs, the hydrophobicity of CBLO decreased the aggregation and/or the swelling of chitosan, resulting in the formation of smaller particles, meaning that the z-average diameter of CS@CBLO NPs decreased with the increasing ratio of CBLO to chitosan. This phenomenon was similar to that seen in other studies [36,37].

PDI is a key parameter showing the quality of the size distribution of nanoparticles in suspensions. With a PDI lower than 0.3 and a single peak in the size distribution curve, it is considered to be monodisperse-sized dispersion [38]. In Figure 3, it can be seen that the highest PDI value was found to be 0.35 for CS NPs, while CS@CBLO NPs had a lower PDI value of 0.22–0.27. The results indicated that CS@CBLO NPs were a monodisperse dispersion with a low variability and no aggregation as compared to CS NPs. In addition, the zeta potential measurement is also shown in Figure 3. The zeta potential of CS NPs showed a positive charge of +36.9 mV contributed by the protonated amino group (NH_3_^+^) of chitosan. On the contrary, the zeta potential of CS@CBLO NPs decreased as the ratio of CBLO to chitosan increased. It has been reported that the zeta potential is related to the number of TPP and chitosan charge groups [39]. With the increasing CBLO, the relative proportion of TPP and chitosan decreased. Moreover, the reason for the decreasing zeta potential may be due to the shielding effect of CBLO reducing the surface charge of chitosan. Several studies have pointed out that when the chitosan encapsulated carvacrol [40], krill oil [36], and eugenol [41], the positively charged surface was reduced with the increasing initial content of the loaded drugs.

### 2.2. Thermogravimetric Analysis

**Figure 4:**
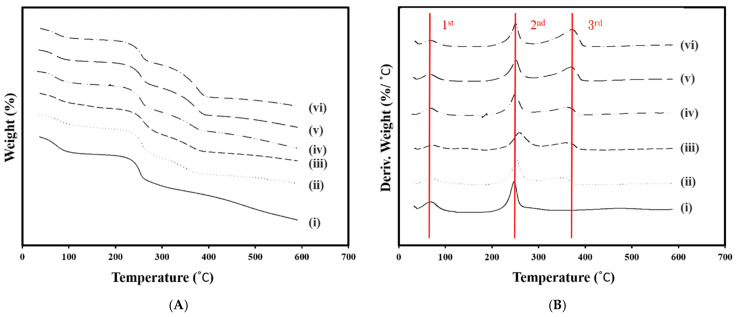
(**A**) TGA and (**B**) DTG thermograms of (**i**) CS NPs and (**ii**)–(**vi**) CS@CBLO NPs prepared using different weight ratios of CBLO to chitosan of (**ii**) 0.25:1, (**iii**) 0.50:1, (**iv**) 0.75:1, (**v**) 1.00:1, and (**vi**) 1.25:1.

TGA is a useful technique for studying the weight change of samples with increasing temperature. The degradation temperature (Td) is the temperature corresponding to the maximum rate of weight loss at each stage. The peak of Td can be clearly observed from the first derivative of the TGA curve with respect to temperature, called derivative thermogravimetry (DTG). As shown in Figure 4A, the weight of CS NPs and CS@CBLO NPs decreased as the temperature increased from 25 to 600 °C. The CS NPs showed two stages of weight loss (Figure 4A-i,B-i). The first and second stages of the weight loss of CS NPs were at temperature range of 31 to 114 °C and 171 to 293 °C, corresponding to water evaporation and chitosan decomposition, respectively. However, CS@CBLO NPs showed three stages of weight loss (Figure 4A-ii–vi,B-ii–vi). Compared to the TGA/DTG of CS NPs (Figure 4A-i,B-i), the third stage weight loss of CS@CBLO NPs in a temperature range of 293 to 415 °C was caused by CBLO decomposition. From the DTG thermograms, CS NPs exhibited two-stage degradation, at 65 °C and 250 °C, respectively (Figure 4B-i). However, the CS@CBLO NPs showed new Td at 368 °C (Figure 4B-ii–vi), corresponding to the Td of CBLO. The results confirmed that the encapsulation of CBLO into CS@CBLO NPs was successful.

### 2.3. Encapsulation Efficiency and Loading Capacity

The TGA/DGT technique can be used for quantitative analysis. The amount of CBLO loaded can be determined by the weight loss of CS@CBLO NPs at temperatures of 293–415 °C (Figure 4A-ii–vi), while the percentage of LC and EE of CBLO is calculated by Eqs. (1) and (2) in Section 3.5. According to the results of TGA, the LC and EE of CBLO are listed in Table 1. When the ratio of CBLO to chitosan increased from 0.25 to 1.25 (*w/w*), the percentage of LC increased from 17.77 to 33.43%. These results indicated that the percentage of LC depended on the initial CBLO concentration, which is consistent with the findings other studies—i.e., that the loading of krill oil, carvacrol, and eugenol in CS NPs is related to the initial concentration of the core material [36,41]. On the other hand, the EE ranged from 25.93% to 50.27%, and the maximum EE of 50.6% was obtained at the ratio of CBLO to chitosan of 0.25 (*w/w*). However, EE decreased with the increased ratio of CBLO to chitosan. The decrease in EE with the increasing of CBLO concentration indicated that CBLO loaded in CS@CBLO NPs is limited because the amount of chitosan is fixed. With an increasing CBLO concentration, the CBLO that can be encapsulated by chitosan gradually reaches saturation, resulting in a decrease in EE.

### 2.4. Characterization XRD and FTIR Spectroscopy

**Figure 5:**
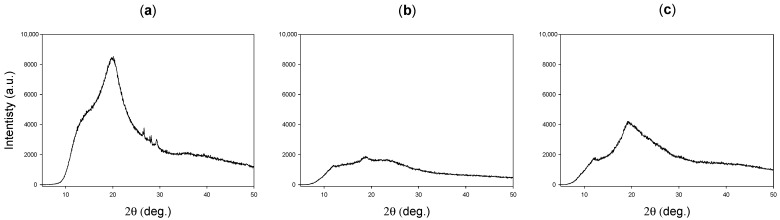
The XRD patterns of (**a**) chitosan powder, (**b**) CS NPs, and (**c**) CS@CBLO NPs.

The crystal structure of chitosan powder, CS NPs, and CS@CBLO NPs was analyzed using the XRD technique. As Figure 5a shows, chitosan exhibits the main diffraction peak at 20.3°, indicating the high degree of crystallinity. After the electrostatic interaction with TPP, no peak was found in the diffractograms of CS NPs (Figure 5b). A quite flat diffraction pattern was obtained, indicating an amorphous structure. The width of the peaks in the XRD pattern is related to the grain size of the crystallites, and the broadened peaks are usually caused by imperfect crystals [42]. Therefore, the broad peak of CS NPs might be caused by ionic gelation with TPP, which did not allow a regular arrangement of the polymer network, leading to its amorphous structure [43]. Compared to chitosan and CS NPs, the characteristic peak of CS@CBLO NPs (Figure 5c) slightly shifted to 18.8° and was markedly sharp, confirming the presence of CBLO within CS NPs. This result also confirmed that the incorporation of CBLO caused a change in the packaging structure of chitosan-TPP.

**Figure 6:**
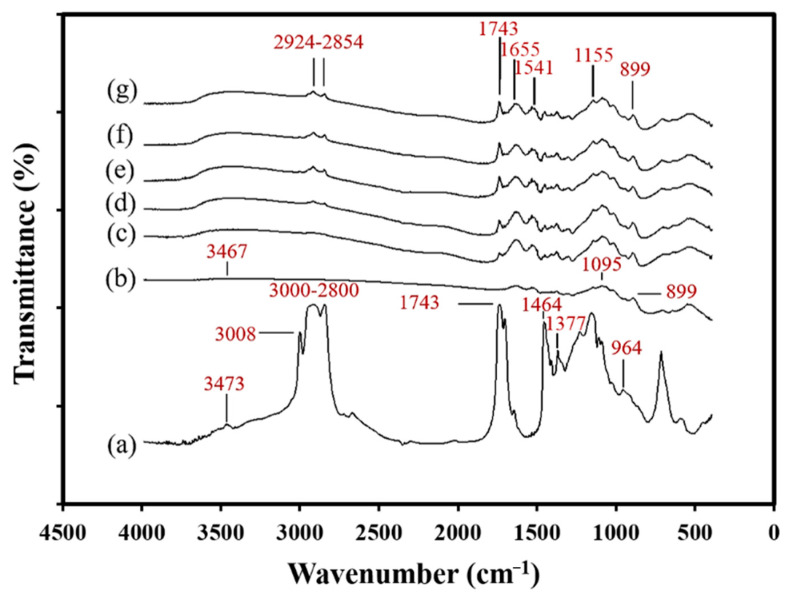
FTIR spectra of (**a**) CBLO, (**b**) CS NPs, and (**c**–**g**) CS@CBLO NPs prepared using different weight ratios of CBLO to chitosan of (**c**) 0.25:1, (**d**) 0.50:1, (**e**) 0.75:1, (**f**) 1.00:1, and (**g**) 1.25:1.

The results of the FTIR showing the characteristic spectra of CBLO, CS NPs, and CS@CBLO NPs are presented in Figure 6. Figure 6a shows the characteristic peaks of CBLO appearing at 3473 cm^−1^ (C=O overtone), 3008 cm^−1^ (=CH stretching), 3000–2800 cm^−1^ (CH stretching), 1743 cm^−1^ (C=O stretching band), 1464 cm^−1^ (–CH_2_–bending), 1377 cm^−1^ (–CH_3_ bending), and 964 cm^−1^ (C=C stretching band). On the other hand, CS NPs shows the characteristic peaks of chitosan and TPP in Figure 6b. The characteristic peaks of CS NPs were found at 3467 cm^−1^ (OH stretching), 1655 cm^−1^ (amide I stretching), 1541 cm^−1^ (amide II stretching), 1155 cm^−1^ (P=O stretching), 1095 cm^−1^ (C–O–C stretching), and 899 cm^−1^ (P-O-P stretching). Compared to CBLO and CS NPs, all the characteristic peaks of both appeared in the FTIR spectra of CS@CBLO NPs (Figure 6c–g), indicating no modification or interaction between the CBLO and chitosan or TPP. In particular, the characteristic peaks of CS@CBLO NPs located at 2924–2854 cm^−1^ (CH stretching), and 1743 cm^−1^ (C=O stretching band), assigned to the methylene and carbonyl group of triglycerides, significantly increased in intensity with an increased ratio of CBLO to chitosan. This result not only reflected the presence of CBLO in CS@CBLO NPs but also showed the content of CBLO in CS@CBLO NPs. Compared to the results of LC in Table 1, the LC of CBLO increased with the increased ratio of CBLO to chitosan. Therefore, the peaks of CH stretching at 2924–2854 cm^−1^ and the C=O stretching band at 1743 cm^−1^ can be used as an indicator to represent the content of CBLO loaded into chitosan nanoparticles. With the analysis of XRD and FTIR, the two-step method through emulsification and ionic gelation is shown to be suitable for encapsulating CBLO in CS@CBLO NPs.

### 2.5. Oxidative Stability

**Figure 7:**
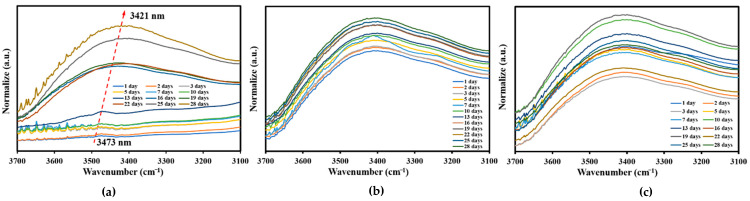
The changes of FTIR spectra at the region around 3400 to 3500 cm^−1^ during storage at room temperature for four weeks: (**a**) CBLO, (**b**,**c**) CS@CBLO NPs prepared using the weight ratios of CBLO to chitosan of (**b**) 1.00:1, and (**c**) 1.25:1.

**Figure 8:**
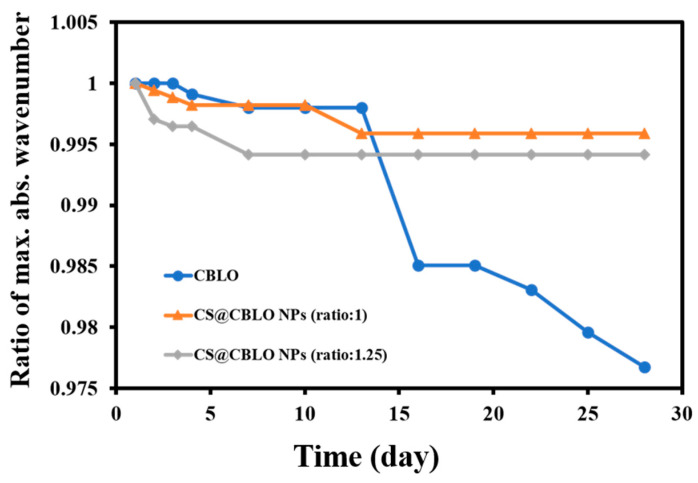
The ratio of maximum absorbance wavenumber in the region of 3400–3500 cm^−1^ during storage at room temperature for four weeks.

**Figure 9:**
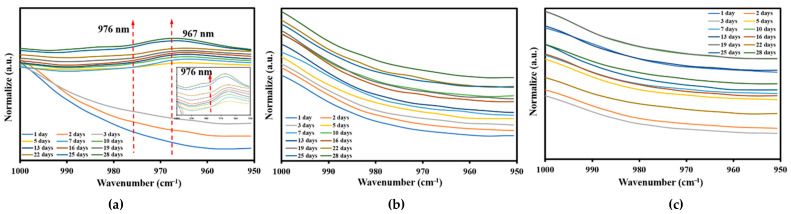
The changes in the FTIR spectra at the wavenumber of 967 and 976 cm^−1^ during storage at room temperature for four weeks: (**a**) CBLO, (**b**,**c**) CS@CBLO NPs prepared using the weight ratios of CBLO to chitosan of (**b**) 1.00:1, and (**c**) 1.25:1.

The anti-oxidation capability to avoid CBLO oxidation is an important indicator for CS@CBLO NPs. CBLO is highly susceptible to oxidation due to its large number of PUFAs. In the early stage of oil oxidation, oxygen directly interacts with conjugated diene to form hydroperoxides [44]. FTIR spectroscopy has been used in the past to identify changes in functional groups in samples that have undergone lipid oxidation [45]. Non-oxidized oils show a narrow weak band in the region of 3400–3500 cm^−1^, with maximum absorbance wavenumbers around 3470 cm^−1^, which are assigned to the overtone of the glyceride ester carbonyl absorption [46]. However, the hydroperoxides generated in the oxidation process cause the maximum absorption to shift to lower wavenumbers [47]. The oxidative stability of CBLO and CS@CBLO NPs during the storage period was observed by FTIR. As Figure 7a shows, the FTIR spectra of CBLO obviously change with the storage time. The maximum absorbance wavenumbers shifted from 3473 to 3421 cm^−1^ as the storage time increased from 1 to 28 d. The peak shift of CBLO was due to the formation of hydroperoxides during storage. In contrast, the FTIR spectra of CS@CBLO NPs (Figure 7b,c) in the region of 3400–3500 cm^−1^ were more stable and without significant shifts or changes. This result indicated that CS@CBLO NPs had a lower hydroperoxide formation during storage compared to CBLO. The ratio of maximum absorbance wavenumber changes in the region of 3400–3500 cm^−1^ during storage can be seen in Figure 8. During the first two weeks of storage, CBLO showed little change, but by day 16 the formation of hydroperoxides caused a significant decrease in the ratio of the maximum absorbance wavenumber. At the same time, the ratio of maximum absorbance wavenumber for CS@CBLO NPs was only slightly decreased, supporting the notion that it had better antioxidative ability. On the other hand, the wavenumber at 967 cm^−1^ was associated with the bending vibrations of CH functional groups of isolated trans-olefins; the increase in trans double bonds during thermal oxidation can be observed by the increasing peak intensities at 967 cm^−1^ [48]. The wavenumbers at 973 and 976 cm^−1^ can be assigned to secondary oxidation products, such as aldehydes or ketones, supporting isolated trans-double bonds [49]. As Figure 9a shows, the peak intensities of CBLO at 967, 973, and 976 cm^−1^ increase with storage time, while the peak intensities of CS@CBLO NPs are almost flat with storage time. These results suggest that CBLO produced more trans double bonds and secondary oxidation products. Several studies have demonstrated that chitosan has good antioxidant properties, especially antioxidant activity, scavenging ability on hydroxyl radicals and chelating ability on ferrous ions [50,51,52]. In this paper, we clearly found that CS@CBLO NPs showed lower lipid hydroperoxides and secondary oxidation products, which might be attributed to chitosan providing good protection against CBLO oxidation.

## 3. Materials and Methods

### 3.1. Materials

Crude cobia liver oil was extracted from cobia liver using homogenization in addition to the sonication method [53]. Briefly, 100 g of cobia liver was homogenized with 1 L hexane using a Polytron PT2100 homogenizer (Kinematica, Littau, Switzerland) equipped with a Polytron PT-DA 2120/2EC probe at 15,000 rpm for 2 min at room temperature, followed by treatment in an ultrasonic bath (40 kHz, Delta D400H, Taipei, Taiwan) for 1 h. The mixture was centrifuged at 3000 rpm for 5 min to remove the solid cobia liver. The supernatant was transferred to a rotary evaporator at 70 °C in order to remove the hexane and recover the oil. The refined cobia liver oil (CBLO) was obtained via degumming, neutralization, and bleaching, according to the procedures described previously [4]. The CBLO containing 24.52% total ω-3 PUFAs (18.85% DHA, 4.25% EPA, and 1.42% α-linolenic acid) was stored at −20 °C until further use. Fatty acid methyl esters of the standards of DHA and EPA and CBLO were prepared via saponification and methylation. The fatty acid composition was analyzed using the GC method by the Center for Aquatic Products Inspection Service, NKUST. cis-4,7,10,13,16,19-Docosahexaenoic acid was purchased from Acros (Fair Lawn, NJ, USA). cis-5,8,11,14,17-Eicosapentaenoic acid was purchased from TCI Co., LT. (Tokyo, Japan). Fatty acid methyl ester standards (Supelco 37 Component FAME Mix, Catalog No. 47885) and BF_3_-methanol reagent (14% BF_3_ in CH_3_OH, *w/v*) were purchased from Sigma-Aldrich (St. Louis, MO, USA). Chitosan (degree of deacetylation of 94.42%) with an average molecular weight of 350 kDa was obtained from Charming & Beauty Co., Ltd. (Taipei, Taiwan). Tween 80 was purchased from Scharlau Chemical Reagent Co., Ltd. (Barcelona, Spain). Acetic acid was purchased from Riedel-de Haën (Seelze, Germany). The sodium tripolyphosphate (TPP) and sodium hydroxide were purchased from Wako Pure Chemical Industries (Osaka, Japan). Unless otherwise specified, all the reagents and chemicals used in this study were of analytical grade. All of the experiments were carried out using double-deionized water (18.2 Ω) using a machine from Merck Millipore.

### 3.2. Fabrication of CS-TPP Encapsulated CBLO Nanoparticles

The CS@CBLO NPs was prepared by a two-step method—i.e., via emulsification and ionic gelation [54]—with some modification. Briefly, chitosan powder was added to 1% (*v/v*) aqueous acetic acid solution at room temperature and stirred gently overnight to prepare the 1.5% (*w/v*) chitosan solution. The chitosan solution was centrifuged at 8000 rpm for 20 min, and the supernatant was collected for further use. Afterwards, 40 mL of chitosan solution was added to 0.5 g of Tween 80 and stirred at 45 °C for 2 h to obtain a transparent solution. The CBLO was gradually dropped into the transparent chitosan solution (40 mL) during homogenization (Polytron PT2100 with PT-DA 2112/2EC probe, Kinematica, Littau, Switzerland) at a speed of 13,000 rpm for 1 min and 16,500 rpm for 2 min to obtain an oil-in-water emulsion. Emulsions with different weight ratios of CBLO to chitosan (0:1, 0.25:1, 0.5:1, 0.75:1, 1:1, and 1.25:1, respectively) were prepared. After emulsification, 40 mL of TPP solution (0.5%, *v/v*) was gradually dropped into the emulsion under continuous stirring for 40 min. The formed particles were harvested using centrifugation at 10,000 rpm for 30 min, then subsequently washed several times with deionized water. Finally, the wet particles were dispersed in 25 mL of water by Q700 sonicator (Qsonica, CT, USA) to produce a homogeneous suspension. The ultrasonication was carried out in an ice bath operated at 30% amplitude for 2 min. The suspensions were immediately freeze-dried at −40 °C for 72 h to obtain the final product, CS@CBLO NPs, and were stored in dry conditions at room temperature.

### 3.3. Morphology of Nanoparticles

SEM was used to observe the morphology of the CS NPs and CS@CBLO NPs. One drop of the sample (50 μg/mL) was placed on a glass plate and dried at room temperature. The dried sample was sputter-coated with gold and then observed through an environmental scanning electron microscope (ESEM; FEI Quanta-200, Brno-Černovice, Czech Republic) under an accelerated voltage of 20 keV. The average particle size was measured from SEM images using an image analyzer (SigmaScan Pro 5, Chicago, IL, USA); in total, about 50 particles were measured.

### 3.4. Particle Size and Zeta Potential Measurement

The z-average diameter, polydispersity index (PDI), and zeta potential of CS NPs and CS@CBLO NPs were measured by dynamic light scattering (DLS) using a Brookhaven 90Plus nanoparticle size analyzer (Brookhaven Instruments, Holtsville, NY, USA). An approximately 2 mg sample was suspended in 5 mL of water using magnetic agitation for mono-dispersion, and then the sample was taken to fill the disposable zeta potential cuvettes for size distribution and surface charge analysis. All the tests were performed in triplicate.

### 3.5. Determination of Encapsulation Efficiency and Loading Capacity

To quantify the CBLO content in CS@CBLO NPs, the TGA/ DTG (derivative thermal gravimetric) techniques were employed for evaluating loading capacity (LC) and encapsulation efficiency (EE). Freeze dried CS NPs and CS@CBLO NPs were, respectively, analyzed by a TGA furnace at 25–600 °C with a heating rate of 10 °C/min under a nitrogen atmosphere. The percentage of weight loss of each composition during thermal decomposition obtained from TGA was used to determine the content of CBLO in CS@CBLO NPs. The equations for the calculation of the loading capacity (Equation (1)) and encapsulation efficiency (Equation (2)) are listed below:

(1) $$\mathrm{LC}=\frac{\mathrm{Weight of loaded CBLO}}{\mathrm{Weight of sample}}\times 100$$

(2) $$\mathrm{EE}=\frac{\mathrm{Weight of loaded CBLO}}{\mathrm{Weight of initial CBLO}}\times 100$$

### 3.6. Characterization using FTIR, TGA and XRD

The FTIR spectra of CBLO, CS NPs, and CS@CBLO NPs were measured by a Horiba FT-730 spectrometer (Kyoto, Japan). A sample of about 6 mg was mixed with 100 mg of KBr and then pressed in a standard device using a pressure of 6000 W to produce 13 mm-diameter pellets. For the CBLO spectral measurement, the liquid sample (~2 μL) was deposited onto a KBr disk. The spectra (4000–400 cm^−1^) were recorded with a resolution of 4 cm^−1^ and 64 scans were performed per sample.

Thermogravimetric analysis (TGA) was performed with a TA2000/2960 thermogravimetric analyzer. Each freeze-dried sample (~5 mg) was placed into the TGA furnace and measurements were carried out under a nitrogen atmosphere with a heating rate of 10 °C/min from 25 to 600 °C.

X-ray diffraction (XRD) patterns of samples were recorded in the scanning mode on a Bruker D8ADVANCE diffractometer operated at a voltage of 40 kV and a current of 30 mA with Cu Kα radiation (λ = 1.5405 Å). The scanning angle (2θ) travelled from 5° to 50° with a scanning speed of 3° min^−1^.

### 3.7. Oxidative Stability

The lipid hydroperoxides, which were the primary oxidation products, were examined by FTIR to evaluate the oxidation stability of CS@CBLO NPs according to the previous literature [46]. The CBLO and CS@CBLO NPs were stored at room temperature for 4 weeks and FTIR measurement was performed every 2 to 3 days. The FTIR spectrum was monitored at a specific wavenumber, around 3500 cm^−1^ to 3400 cm^−1^ for hydroperoxides (ROOH), 967 cm^−1^ for trans double bond and 973 or 976 cm^−1^ for secondary oxidation products, such as aldehydes or ketones supporting isolated trans-double bonds, in order to observe the oxidation stability of CBLO with or without encapsulation. The ratio of maximum absorbance wavenumber in the region of 3400–3500 cm^−1^ was defined as fn/fo. The fo was the initial maximum absorbance wavenumber and fn was the maximum absorbance wavenumber at each time point.

## 4. Conclusions

In this work, CS-TPP encapsulated CBLO nanoparticles (CS@CBLO NPs) were successfully fabricated using a two-step method via emulsification and ionic gelation. The particle size and its structure were revealed to be 347 nm and 174–456 nm, respectively, by scanning electron microscopy (SEM) and dynamic light scattering (DLS). The loading capacity (LC) and encapsulation efficiency (EE) of CBLO in CS@CBLO NPs were determined according to the TGA/DTG result at the degradation temperature of 368 °C. The LC and EE values were shown to be about 17.77~33.43% and 25.93~50.27%, respectively, when the ratio of CBLO to chitosan was in the range of 0.25–1.25. The oxidative stability of CS@CBLO NPs evaluated by FTIR showed that the CS@CBLO NPs could effectively prevent CBLO oxidation. The results demonstrated that the strategy of encapsulation using chitosan can effectively protect high-sensitivity materials from metamorphism. The CS@CBLO NPs combined two healthy ingredients, chitosan and fish oil, a kind of nano powdered fish oil; it can be conveniently used in various foods as a fortification of DHA and EPA to enhance their usage in the food and pharmaceutical industries.

## Figures and Tables

**Table 1:** The effect of different ratios of CBLO to chitosan on the loading capacity and encapsulation efficiency.

CBLO: Chitosan (*w/w*)	LC (%)	EE (%)
0.25:1.00	17.77 ± 0.09	50.27 ± 0.30
0.50:1.00	17.97 ± 0.02	29.53 ± 0.19
0.75:1.00	22.03 ± 0.22	25.93 ± 1.14
1.00:1.00	30.16 ± 0.48	29.40 ± 0.33
1.25:1.00	33.43 ± 0.37	28.47 ± 0.17

## Data Availability

Data is contained within the article.
